# Determinants of Abnormal Pulmonary Vasodilatory Response With Exercise in HFpEF: Pulmonary Vascular‐Left Atrial Axis Abnormalities

**DOI:** 10.1002/cph4.70229

**Published:** 2026-07-26

**Authors:** Mariana Garcia‐Arango, Angela Kristo, Ahmed El Shaer, Yimin Chen, Aditya Sahai, Anas Abed, Wanxin Tu, Soham Ghosh, Babak Tehrani, Shannon Heffernan, Amir Esmaeeli, Tashfeen Javaid, Ran Tao, Naga Dharmavaram, James Runo, Alejandro Roldán‐Alzate, Adam D. Gepner, Matthew Kalscheur, Colleen M. Witzenburg, Ryan J. Tedford, Farhan Raza

**Affiliations:** ^1^ Department of Medicine‐Cardiovascular Division University of Wisconsin‐Madison Madison Wisconsin USA; ^2^ School of Medicine and Public Health University of Wisconsin‐Madison Madison Wisconsin USA; ^3^ Department of Internal Medicine University of Wisconsin‐Madison Madison Wisconsin USA; ^4^ Department of Statistics University of Wisconsin‐Madison Madison Wisconsin USA; ^5^ Fatima Jinnah Medical University Lahore Pakistan; ^6^ Department of Medicine‐Pulmonary and Critical Care Division University of Wisconsin‐Madison Madison Wisconsin USA; ^7^ Department of Radiology University of Wisconsin‐Madison Madison Wisconsin USA; ^8^ Department of Mechanical Engineering University of Wisconsin‐Madison Madison Wisconsin USA; ^9^ Department of Medicine – Division of Cardiovascular Medicine William S. Middleton Memorial Veteran's Hospital Madison Wisconsin USA; ^10^ Department of Biomedical Engineering University of Wisconsin‐Madison Madison Wisconsin USA; ^11^ Department of Medicine‐Cardiovascular Division Medical University of South Carolina Charleston South Carolina USA

**Keywords:** atrial fibrillation, COPD, HFpEF, pulmonary hypertension, pulmonary vascular resistance

## Abstract

**Background:**

Inability to decrease pulmonary vascular resistance (PVR) with exercise may lead to right ventricular failure. In this two‐step study, we define an unfavorable exercise PVR response among a broad cohort and then determine its high‐risk correlates in HFpEF.

**Methods:**

164 participants (80 HFpEF, 57 pre‐capillary PH, 27 non‐cardiac dyspnea) underwent invasive cardiopulmonary exercise test. Dichotomous groups were created with a stepwise approach: “unfavorable exercise PVR” (*n* = 85, HFpEF = 46) defined as exercise PVR > 1.74Woods unit (WU) and ∆PVR decrease with exercise of < 22%, the remainder of the cohort was labeled as “favorable exercise PVR” (*n* = 79, HFpEF = 34). Stepwise approach included: physiological groups (tertiles) using exercise PVR cutoff = 1.74 WU and median approach with ∆PVR decrease with exercise.

**Results:**

In unfavorable (vs. favorable) PVR HFpEF subgroups, rest PVR = 3.4 ± 2.0 vs. 2.9 ± 2.0WU, exercise PVR = 3.7 ± 2.6 vs. 2.0 ± 1.3WU, and ∆PVR = +11% ± 39% vs. −26% ± 22%. Correlates of unfavorable exercise PVR with univariate regression were: atrial fibrillation, COPD, increased LAVI, lower TAPSE, and lower TAPSE/PASP. Multivariate model (including clinical data: age, sex, BMI) revealed that atrial fibrillation (β‐estimate = 1.93, *p* = 0.003) and COPD (β‐estimate = 1.74, *p* = 0.03) were significant. However, with multivariate model (including LAVI and TAPSE), COPD remained significant (β‐estimate = 3.26, *p* = 0.02), while atrial fibrillation (β‐estimate = 1.60, *p* = 0.06) and LAVI (β‐estimate = 0.05, *p* = 0.09) approached significance. The unfavorable versus favorable exercise PVR HFpEF subgroups had similar biventricular morphology by cardiac MRI (*p* < 0.30).

**Conclusions:**

Compared to traditional cardiometabolic comorbidities, COPD and atrial fibrillation are uniquely linked to pulmonary vascular remodeling in HFpEF. The impact of these two comorbidities on pulmonary vascular‐left atrial axis is highlighted by the shared biventricular morphology among HFpEF subgroups.

## Graphical abstract

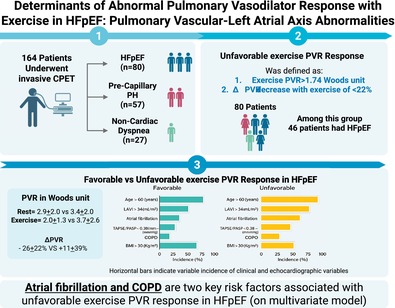

## 1 Introduction

Pulmonary hypertension (PH) is universally defined by mean pulmonary artery pressure (mPAP) > 20 mmHg at rest, though it has multiple distinct hemodynamic phenotypes (Humbert et al. 2022; Vaidya et al. 2020; Raza et al. 2017). Pulmonary vascular remodeling in PH, quantified with pulmonary vascular resistance (PVR), leads to right ventricular (RV) failure and increased mortality in different PH phenotypes, such as pre‐capillary PH and heart failure with preserved ejection fraction (HFpEF) (Maron et al. 2020; Vallerand et al. 2019; Borlaug et al. 2022; Lai et al. 2019). Under normal conditions, pulmonary circulation vasodilates with exercise along with a decrease in PVR, and the inability to appropriately vasodilate indicates advanced pulmonary vascular disease (Borlaug et al. 2022; Malhotra, Bakken, et al. 2016; Lewis et al. 2013; Kovacs et al. 2012). The behavior of the pulmonary vasculature with exercise can be tested non‐invasively (e.g., echocardiogram, cardiopulmonary exercise test: CPET) or with invasive exercise hemodynamics, which can provide early diagnosis and mechanistic insights into different PH phenotypes (Vaidya et al. 2020; Raza et al. 2017; Raza, Dharmavaram, et al. 2022). However, the complexity of defining an impaired exercise PVR response is challenging as there is no standardized definition of a normal response. The need to define abnormal exercise response and characterize pulmonary vascular disease is highest in HFpEF, where it significantly impacts mortality and may influence the suitability of novel therapies, for example, atrial septal devices (Borlaug et al. 2022; Vanderpool et al. 2018; Caravita et al. 2023).

Abnormal PVR responses and blunted cardiac output responses with exercise have been reported in HFpEF and are associated with adverse cardiovascular outcomes (Shah et al. 2022; Caravita et al. 2023). Another recent study with rest‐only hemodynamics suggests that left atrial enlargement and atrial fibrillation are also correlated with elevated PVR in left heart failure patients (left ventricular ejection fraction: LVEF ≥ 40%) (Kelly et al. 2023). While these studies add to growing evidence to define correlates of pulmonary vascular disease in HFpEF, a broader definition of a quantifiable poor exercise PVR response and its correlates (high‐risk patient characteristics and morphological cardiac features) can lead to early targeted management of any patient with exercise intolerance and suspected PH. The current study aims to define an unfavorable PVR response with exercise using a stepwise approach among a broad cohort (varying PH phenotypes and a control group) and to determine correlates of this unfavorable exercise PVR in HFpEF. The findings from this study may lead to further hypothesis generation towards underlying biological mechanisms to understand pulmonary vascular disease in HFpEF.

## 2 Methods

The data that support the findings of this study are available from the corresponding author upon reasonable request.

### 2.1 Study Population

This retrospective study included a cohort of participants who presented to the Pulmonary Hypertension clinic for worsening dyspnea (New York Heart Association class II‐III). The cohort consists of 164 consecutive patients who were referred for a clinically indicated invasive exercise hemodynamic study (right heart catheterization with CPET) to define and diagnose subclinical PH or phenotype PH. All participants had LVEF ≥ 50%. Based on 2022 guidelines (Humbert et al. 2022), three hemodynamic groups were defined based on rest and exercise hemodynamics:

1. HFpEF (*n* = 80): rest mPAP > 20 mmHg and pulmonary artery wedge pressure (PAWP) > 15 mmHg, and among participants with rest mPAP ≤ 20 mmHg, exercise hemodynamics were abnormal (PAWP/CO slope ≥ 2.0 mmHg/L.min^−1^).
2. Pre‐capillary PH (*n* = 57): rest mPAP > 20 mmHg, PAWP ≤ 15 mmHg and PVR > 2 Woods units (WU), and among participants with rest mPAP ≤ 20 mmHg, exercise hemodynamics were abnormal (mPAP/CO slope ≥ 3.0 mmHg/L.min^−1^, and PAWP/CO slope < 2.0 mmHg/L.min^−1^).
3. Non‐cardiac dyspnea (NCD) (*n* = 21): rest mPAP ≤ 20 mmHg, mPAP/CO slope with exercise < 3.0 mmHg/L·min^−1^ and PAWP/CO slope with exercise < 2.0 mmHg/L.min^−1^. Participants with undefined PH with rest mPAP 20–25 mmHg were also included in this group if rest PAWP ≤ 15 mmHg, rest PVR ≤ 2 WU and exercise metrics were normal (mPAP/CO slope < 3.0 mmHg/L·min^−1^ and PAWP/CO slope < 2.0 mmHg/L.min^−1^).

All participants had complete resting transthoracic echocardiographic evaluation, six‐minute walk test, and clinical evaluation by a PH clinician. Exclusion criteria included: supplemental oxygen requirements or reported SpO2 < 92% on 6‐min walk test (before invasive CPET), serum creatinine > 2.5 mg/dL and ≥ moderate valvular disease. The institutional review board approved this study. The study was compliant with the guidelines of the Declaration of Helsinki.

### 2.2 Exercise Hemodynamic Study

All participants underwent invasive exercise hemodynamic evaluation including an exercise right heart catheterization and expired gas analysis (CPET) on a semi‐recumbent ergometer, as described previously (Raza, Dharmavaram, et al. 2022; Kozitza et al. 2022; Le et al. 2022; Tao et al. 2024; Raza, Kozitza, et al. 2022; El Shaer et al. 2024; Zampierollo‐Jaramillo et al. 2024). Briefly, right heart catheterization was performed with a balloon‐tipped catheter through a 7‐Fr venous sheath via internal jugular access. A 4‐Fr radial arterial catheter was placed selectively in participants with SpO2 < 95% previously on a six‐minute walk test or participants without a six‐minute walk test. The breath‐by‐breath expired gas exchange analysis was performed with an Ultima CardiO2 MedGraphics metabolic cart, which was connected to participants via a mouthpiece and nose clip to avoid air leak. Cardiac output was measured at all stages with direct Fick principle. Hemodynamic data were acquired at rest, different stages of exercise (every 20–25 watts, only peak‐exercise data reported), and recovery (2‐min post‐exercise). Specific CPET metrics were recorded: peak oxygen consumption (VO_2_), slope of minute ventilation/carbon dioxide production (V_E_/VCO_2_), and end‐tidal carbon dioxide from rest‐to‐exercise (ETCO_2_).

### 2.3 Cardiac Magnetic Resonance Imaging (MRI)

Analysis of cardiac MRI images was performed using a commercially available software (cvi42, version 5.6.6; Circle Cardiovascular Imaging Inc). Ventricular volumes were calculated from short‐axis, cine‐balanced, SSFP series. Three slices from the LV and the RV (base, mid, and apex) short‐axis and long‐axis cine MRI scans were used to analyze longitudinal, circumferential, and radial dimensions and strain as a function of time, including average peak strain, as previously described (Raza, Kozitza, et al. 2022; Sato et al. 2021). Additional metrics of septal angle and epicardial fat were also acquired from short‐axis cine MRI scans (Saunders et al. 2022; Johns et al. 2018; van Woerden et al. 2021). Atrial volumes and strain were acquired in 4‐chamber and 2‐chamber apical views (Cau et al. 2022).

### 2.4 Stepwise Approach to Define *Unfavorable* Exercise PVR Among Overall Cohort (Three Hemodynamic Groups)

**FIGURE 1:**
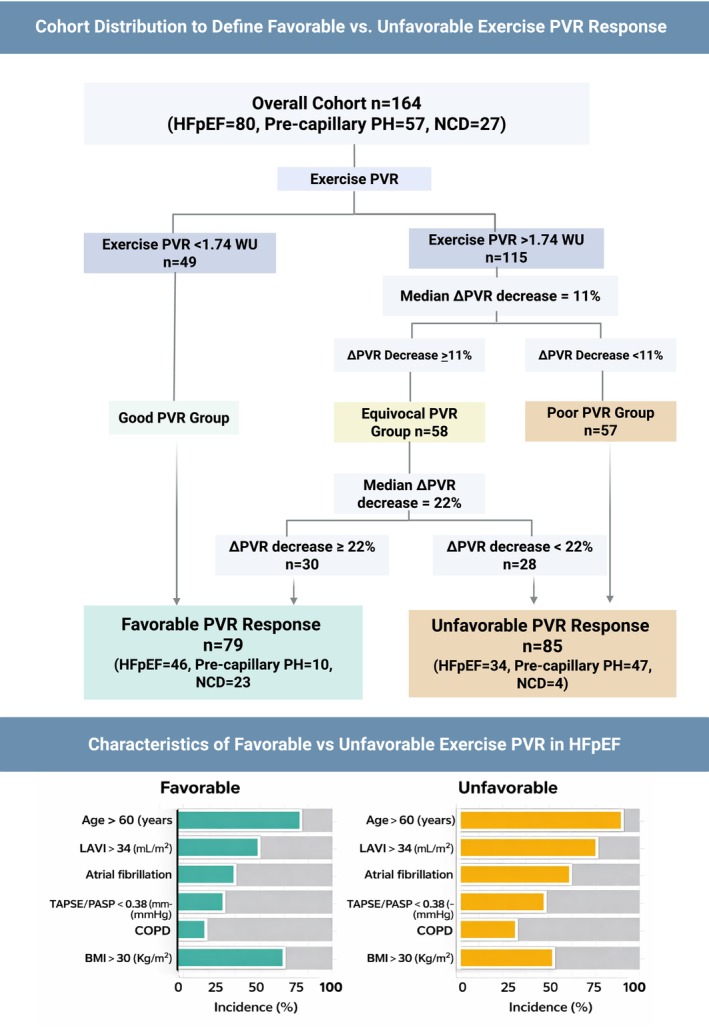
Flowchart of Cohort Distribution to define Favorable vs. Unfavorable PVR response with Exercise. Characteristics of Favorable and Unfavorable Pulmonary Vascular Vasodilatory Response In HFpEF (created with Biorender). PVR, pulmonary vascular resistance; HFpEF, heart failure with preserved ejection fraction; PH, pulmonary hypertension; NCD, non‐cardiac dyspnea; LAVI, left atrial volume index; TAPSE, tricuspid annular plane systolic excursion; PASP, pulmonary artery systolic pressure; COPD, chronic obstructive pulmonary disease; BMI, body mass index.

Figure 1 summarizes the stepwise approach to define an unfavorable PVR response with exercise. The stepwise approach was based on exercise PVR cutoff of > 1.74 WU (based on prior literature (Borlaug et al. 2022; Caravita et al. 2023; Shah et al. 2022)) and median of ∆PVR (rest‐to‐exercise). The rationale for this two‐step approach was to be as comprehensive and inclusive as possible in defining exercise PVR response. For example, exercise PVR ≤ 1.74 Woods unit (WU) group captures exercise vasodilatory response of participants with relatively healthy pulmonary circulation and includes the majority of NCD (control group) along with a significant proportion of HFpEF cohort, albeit pre‐capillary PH participants are excluded. The second step of median ∆PVR includes the majority of the PH participants (HFpEF and pre‐capillary PH) and captures exercise vasodilatory response of participants with diseased pulmonary circulation.

### 2.5 Statistical Analysis

Baseline characteristics and PVR response characteristics were presented as mean ± SD for continuous variables and as frequencies and proportions (%) for categorical variables. For continuous variables, a comparison among groups was conducted using the analysis of variance (ANOVA) test (or Wilcoxon rank sum test for non‐normal distribution). Differences in categorical data were assessed using the *χ* ^2^ statistic or Fisher exact test, as appropriate. To determine which parameters correlate with an unfavorable PVR response with exercise, univariate and multivariate logistic regression analyses were used. Three hemodynamic groups (HFpEF, pre‐capillary PH, and NCD) were taken as fixed categorical variables without interaction in the regression models. All analyses were performed using R (version 4.1).

## 3 Results

### 3.1 Clinical and Echocardiographic Features of HFpEF, Pre‐Capillary PH and NCD Groups

The overall clinical and general characteristics are presented in Table 1. The HFpEF group was older, had equal sex distribution (46% female) and a higher comorbidity burden (e.g., hypertension, atrial fibrillation, sleep apnea). The pre‐capillary PH group had higher incidence of Scleroderma. Spirometry metrics did not differ statistically among groups (HFpEF, pre‐capillary PH, NCD): forced vital capacity, FVC (%): 79 ± 19, 88 ± 22, 100 ± 17, forced expiratory volume in 1 s, FEV1 (%): 75 ± 18, 83 ± 23, 99% ± 22, and diffusion capacity of the lungs for carbon monoxide, DLCO (%): 64 ± 19, 58 ± 22, 89 ± 21.

**TABLE 1:** Clinical, echocardiographic and hemodynamics features of HFpEF, pre‐capillary PH and NCD groups.

Variables	HFpEF (*n* = 80)	Pre‐capillary PH (*n* = 57)	NCD (*n* = 27)	*p*
Age (years)	66.3 ± 12.6	65.0 ± 11.9	60.0 ± 13.9	0.08
Female sex, *n* (%)	37 (46)	35 (61)	17 (63)	0.13
BMI (kg/m^2^)	34.5 ± 8.4	27.9 ± 6.4	30.4 ± 5.6	< 0.001
BNP (pg/mL)	202 ± 220	174 ± 259	48 ± 40	0.056
6MWD (meter)	259 ± 91	283 ± 113	372 ± 115	0.004
Comorbidities				
Diabetes, *n* (%)	26 (33)	16 (28)	5 (19)	0.38
HTN, *n* (%)	70 (88)	35 (61)	13 (48)	< 0.001
COPD, *n* (%)	13 (16)	9 (16)	3 (11)	0.92
CAD, *n* (%)	29 (37)	17 (30)	2 (7)	0.02
Atrial fibrillation, *n* (%)	37 (46)	9 (16)	4 (15)	< 0.001
Scleroderma, *n* (%)	5 (6)	18 (32)	3 (11)	< 0.001
OSA, *n* (%)	38 (49)	18 (32)	10 (37)	0.12
Echocardiographic				
LVEF (%)	61 ± 6	61 ± 6	60 ± 6	0.93
LVMI (g/m^2^)	94.1 ± 43.4	84.2 ± 45	74.5 ± 26.8	0.14
LAVI (mL/m^2^)	40.5 ± 14.5	27.4 ± 14.2	30.2 ± 13.6	< 0.001
E/E' Average	12.4 ± 7.7	11.5 ± 7.6	8.4 ± 3.1	0.15
TAPSE/PASP (mm/mmHg)	0.45 ± 0.23	0.42 ± 0.24	0.80 ± 0.25	< 0.001
Epicardial fat (mm)	6.3 ± 2.6	5.3 ± 2	5.4 ± 3.0	0.07
Rest Hemodynamics				
Heart rate (beats/min)	70 ± 13	71 ± 11	77 ± 10	0.04
mPAP (mmHg)	34 ± 10	39 ± 11	20 ± 2	< 0.001
PAWP (mmHg)	18 ± 4	13 ± 2	12 ± 2	< 0.001
Direct Fick CO (L/min)	5.4 ± 1.4	4.6 ± 1.1	5.9 ± 1.1	< 0.001
SVI (mL/m^2^)	37.1 ± 9.4	35.6 ± 8.7	40.4 ± 7.2	0.09
PVR (woods units)	3.2 ± 2	6.0 ± 3.2	1.4 ± 0.6	< 0.001
Peak exercise hemodynamics				
Heart rate (beats/min)	96 ± 23	103 ± 19	118 ± 19	< 0.001
mPAP (mmHg)	52 ± 12	59 ± 14	31 ± 5	< 0.001
PAWP (mmHg)	31 ± 6	19 ± 3	18 ± 3	< 0.001
Direct Fick CO (L/min)	9.1 ± 3.3	8.4 ± 2.4	12.5 ± 3.1	< 0.001
SVI (mL/m^2^)	44.2 ± 12.2	40.6 ± 10.7	54.3 ± 10.3	< 0.001
PVR (woods units)	2.8 ± 2.1	5.9 ± 4.0	1.2 ± 0.5	< 0.001
∆ Rest‐to‐exercise				
∆ PVR	−0.4 ± 1.1	−0.1 ± 2.2	−0.3 ± 0.3	0.55
PVR change (%)	−10 ± 35	−6 ± 30	−18 ± 22	0.037
mPAP/CO slope (mmHg/L.min^−1^)	8.5 ± 6.6	7.7 ± 5.2	1.8 ± 0.8	< 0.001
PAWP/CO slope (mmHg/L.min^−1^)	6.2 ± 6	1.3 ± 0.5	1.0 ± 0.6	< 0.001
Cardiopulmonary exercise test				
Peak Watts	58 ± 32	56 ± 30	96 ± 46	< 0.01
Peak VO_2_ (mL/kg.min^−1^)	13.9 ± 4.9	14.1 ± 4.8	19.5 ± 6.4	< 0.001
Peak VO_2_%predicted	51 ± 15	54 ± 17	68 ± 22	< 0.001
OUES (mL/min/Log[L/min])	1.25 ± 0.40	1.10 ± 0.39	1.51 ± 0.58	0.001
V_E_/V_CO2_ slope	38.2 ± 10.9	39.80 ± 10.1	34.5 ± 9.8	0.132
∆ETCO_2_ (mmHg)	−0.6 ± 2.4	−0.9 ± 2.9	+1.3 ± 3.2	0.02

Abbreviations: 6MWD, six‐minute walk distance; BMI, body mass index; BNP, brain natriuretic peptide; BNP, B‐type natriuretic peptide; CAD, coronary artery disease; CO, cardiac output; COPD, chronic obstructive pulmonary disease; E/E', ratio of early diastolic mitral blood flow velocity to mitral annulus velocity; HFpEF, heart failure with preserved ejection fraction; HTN, hypertension; LAVI, left atrial volume index; LVEF, left ventricular ejection fraction; LVMI, left ventricular mass index; mPAP, mean pulmonary artery pressure; mPAP/CO, Ratio of Mean Pulmonary Artery Pressure to Cardiac Output; NCD, noncardiac dyspnea; OSA, obstructive sleep apnea; PASP, pulmonary arterial systolic pressure; PAWP, pulmonary artery wedge pressure; PAWP/CO, Ratio of Pulmonary Artery Wedge Pressure to Cardiac Output; PH, pulmonary hypertension; PVR, pulmonary vascular resistance; SVI, stroke volume index; TAPSE, tricuspid annular plane systolic excursion.

On echocardiogram, the HFpEF group had increased left ventricular mass index, left atrial volume index (LAVI), diastolic dysfunction, and epicardial fat. An estimate of RV:PA coupling, the tricuspid annular plane systolic excursion (TAPSE) to pulmonary artery systolic pressure ratio, was equally impaired in HFpEF and pre‐capillary PH groups.

### 3.2 Hemodynamic Features of HFpEF, Pre‐Capillary PH and NCD Groups

As expected with rest hemodynamics, the HFpEF group had elevated PAWP, the pre‐capillary PH group had elevated PVR, and the NCD group had normal hemodynamics (Table 1). At peak exercise, the HFpEF group had a blunted heart rate and severely elevated PAWP (Table 1). Compared to the HFpEF and pre‐capillary PH groups, the NCD group doubled their cardiac output at peak exercise. Overall, PVR decreased with exercise in all groups with no statistically significant difference in ∆PVR among the three groups (Table 1).

### 3.3 Definition of *Unfavorable* Exercise PVR Response Using Stepwise Approach

As summarized in Figure 1, a stepwise approach led to the definition of unfavorable exercise PVR response as exercise PVR > 1.74 WU and a decrease in PVR (from rest‐to‐exercise) of < 22%.

As a first step, the overall cohort was divided into three physiological groups (or tertiles). First group (*n* = 49), labeled as a “*good PVR*”, was defined per current literature as exercise PVR ≤ 1.74 WU (Borlaug et al. 2022; Caravita et al. 2023; Shah et al. 2022). In the rest of the cohort with exercise PVR > 1.74 WU (*n* = 115), based on median ∆PVR (−11%), “*equivocal PVR*” (*n* = 58) and “*poor PVR*” (*n* = 57) groups were defined.

As a second step, to create dichotomous groups, the “*equivocal PVR*” group was further subdivided (based on median ∆PVR = −22% within this group). Participants with a ∆PVR decrease with exercise of > 22% (more negative) were added to the “*good PVR*” group to create a “*favorable PVR response*” group (*n* = 79), while the participants with a ∆PVR decrease with exercise of < 22% were added to “*poor PVR*” group to create an “*unfavorable PVR response*” group (*n* = 85). Notably, HFpEF participants were nearly equally distributed among the two PVR response categories, which indicates heterogenous PVR response among a diverse HFpEF population (see Figure 1).

### 3.4 Features of *Unfavorable* Exercise PVR Response in HFpEF

HFpEF participants with unfavorable exercise PVR response trended towards an older population with lower BMI and poor oxygen uptake slope (Table 2, Figure 1). These patients had an increased incidence of atrial fibrillation, chronic obstructive pulmonary disease (COPD), increased left atrial size, worse RV:PA coupling (TAPSE/PASP), and poor cardiac output augmentation with exercise. As expected from predefined hemodynamic criteria in unfavorable (vs. favorable) PVR groups, ∆PVR (rest‐to‐exercise) increased by +11 ± 39% (vs. decrease by −26% ± 22%, *p* < 0.0001). On survival analysis, there was a non‐significant trend towards worse survival in the unfavorable PVR group compared with the favorable PVR group (log‐rank *p* = 0.113), with a hazard ratio of 2.01 (95% CI, 0.90–4.45) (Figure S1).

**TABLE 2:** Features of HFpEF favorable versus unfavorable exercise PVR response sub‐groups.

Variable	HFpEF group with favorable PVR response (*n* = 46) exercise PVR ≤ 1.74 WU; Or exercise PVR > 1.74 WU and ∆PVR decrease > 22%	HFpEF group with unfavorable PVR response (*n* = 34) exercise PVR > 1.74 WU and ∆PVR decrease < 22%	*p*
IpcPH/CpcPH	22/24	5/29	
Age (years)	64.3 ± 14	69.1 ± 9.9	0.08
Female sex, *n* (%)	21 (46)	16 (47)	0.95
BMI (kg/m^2^)	35.3 ± 8.4	33.5 ± 8.4	0.36
BNP (pg/mL)	126 ± 172	288 ± 239	0.01
6MWD (meter)	268 ± 96	249 ± 86	0.49
Diuretic use, *n* (%)	22 (48%)	19 (56%)	0.26
Comorbidities			
Diabetes, *n* (%)	15 (33)	11 (32)	0.66
HTN, *n* (%)	40 (87)	30 (88)	0.32
COPD, *n* (%)	3 (7)	10 (29)	0.012
CAD, *n* (%)	18 (40)	11 (32)	0.64
Atrial fibrillation, *n* (%) [paroxysmal/permanent]	14 (30) [4/10]	23 (68) [7/16]	< 0.001
Scleroderma, *n* (%)	3 (7)	2 (6)	0.31
OSA, *n* (%)	23 (52)	15 (44)	0.48
H_2_FPEF score	4.8 ± 1.8	5.6 ± 2	0.08
Echocardiographic			
LVMI (g/m^2^)	97.3 ± 42.6	89.3 ± 45.3	0.47
LAVI (mL/m^2^)	36.9 ± 14.8	45.7 ± 12.9	0.03
E/E' average	12.3 ± 6.1	12.4 ± 10.1	0.97
RV FAC (%)	35 ± 10	34 ± 8	0.75
TAPSE (mm)	21.5 ± 8	17.4 ± 4.3	0.02
TAPSE/PASP	0.51 ± 0.27	0.35 ± 0.11	0.01
Eccentricity Index	1.08 ± 0.29	1.11 ± 0.31	0.70
Epicardial fat (mm)	6.2 ± 2.9	6.4 ± 2.3	0.81
Rest hemodynamics			
Heart rate (beats/min)	71 ± 13	70 ± 13	0.68
mPAP (mmHg)	33 ± 10	35 ± 10	0.37
PAWP (mmHg)	18 ± 3	19 ± 4	0.13
Direct Fick CO (L/min)	5.8 ± 1.4	4.9 ± 1.1	0.002
SVI (mL/m^2^)	38.9 ± 10.5	34.5 ± 6.8	0.03
PVR (woods units)	2.9 ± 2.0	3.4 ± 2.0	0.25
Peak exercise hemodynamics			
Heart rate (beats/min)	100 ± 22	90 ± 24	0.05
mPAP (mmHg)	49 ± 11	56 ± 14	0.01
PAWP (mmHg)	31 ± 6	32 ± 7	0.51
Direct Fick CO (L/min)	10.3 ± 3.4	7.1 ± 2.0	< 0.001
SVI (mL/m^2^)	48.5 ± 13.3	38.2 ± 7.2	< 0.001
PVR (woods units)	2.0 ± 1.3	3.7 ± 2.6	0.001
∆ PVR, % rest‐to‐exercise	−26 ± 22	+11 ± 39	< 0.001
Cardiopulmonary exercise test			
Peak watts	62 ± 34	54 ± 30	0.27
Peak VO_2_ (mL/kg.min^−1^)	14.6 ± 5.5	13.0 ± 3.8	0.14
Peak VO_2_ (% predicted)	51 ± 15	50 ± 15	0.78
Peak O_2_ pulse (mL/beat)	10.4 ± 2.7	10.6 ± 7.6	0.85
OUES (mL/min/Log[L/min])	1.32 ± 0.42	1.16 ± 0.36	0.09
V_E_/VCO_2_ slope	35.9 ± 7.8	39.7 ± 12.5	0.12
∆ETCO_2_ (mmHg)	−0.4 ± 2.4	−1.1 ± 2.7	0.22

Abbreviations: ∆ETCO_2_, change in end‐tidal carbon dioxide; ∆PVR, change in pulmonary vascular resistance; 6 MWD, 6‐minute walk distance; BMI, Body Mass Index; BNP, B‐type Natriuretic Peptide; COPD, chronic obstructive pulmonary disease; CpcPH, combined pre‐ and post‐ capillary pulmonary hypertension; CPET, cardiopulmonary exercise testing; E/E' average, ratio of early diastolic mitral inflow velocity to mitral annular early diastolic velocity; HTN, hypertension; IpcPH, isolated post‐capillary pulmonary hypertension; LAVI, Left atrial volume index; LVMI, left ventricular mass index; OUES, oxygen uptake efficiency slope; Peak O_2_ pulse, peak oxygen pulse; Peak VO_2_, peak oxygen consumption; PVR, pulmonary vascular resistance; TAPSE, Tricuspid Annular Plane Systolic Excursion; VE/VCO_2_ slope, ventilation‐to‐carbon dioxide production slope.

### 3.5 Cardiac‐MRI Based Morphology of HFpEF Sub‐Groups: Similar Ventricular Features, Abnormal Left Atrium

**FIGURE 2:**
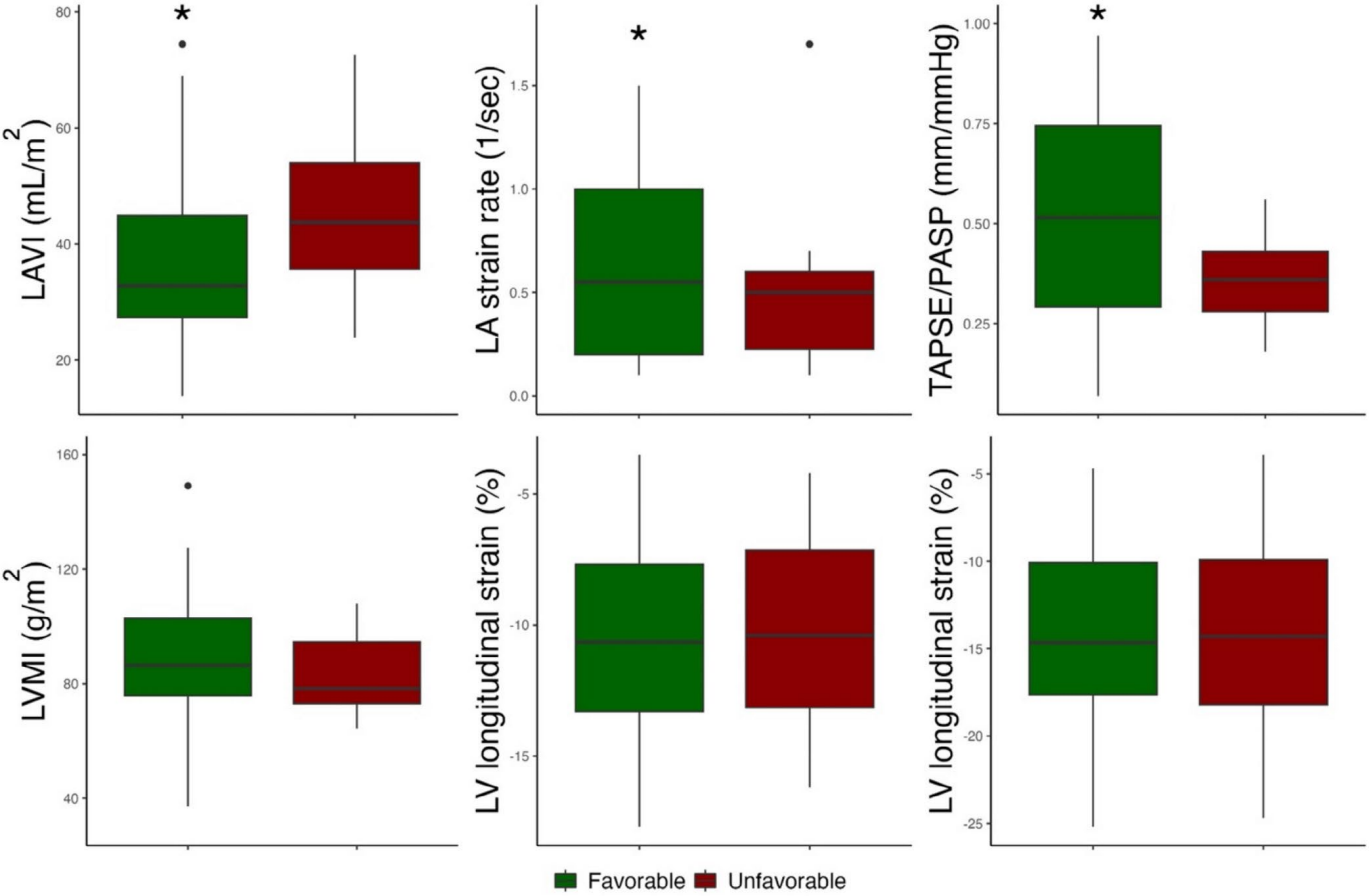
Abnormal pulmonary vascular vasodilatory response in HFpEF related to atrial fibrillation, left atrial myopathy, and COPD (despite similar left ventricular structure). Box‐and‐whisker plots comparing echocardiographic (LAVI, TAPSE/PASP) and cardiac magnetic resonance imaging characteristics of the HFpEF subgroups. *p < 0.05. HFpEF, heart failure with preserved ejection fraction; LAVI, left atrial volume index; TAPSE, tricuspid annular plane systolic excursion; PASP, pulmonary artery systolic pressure; COPD, chronic obstructive pulmonary disease; BMI, body mass index; LVMI, left ventricular mass index; RV, right ventricle.

In a sub‐cohort of HFpEF participants (*n* = 48) with available cardiac MRI (within 12‐months of exercise hemodynamic study), deeper morphological characterization was performed (Table 3, Figure 2). Interestingly, detailed characterization of left ventricular and right ventricular structure (mass, volume, feature‐tracking strain) revealed that ventricular structure was similar (statistically not significant) among HFpEF subgroups with favorable vs. unfavorable PVR response. The only notable difference was a reduced LA strain rate in the unfavorable PVR sub‐group, while there was a trend towards lower LA longitudinal strain and ejection fraction.

**TABLE 3:** Cardiac MRI features of HFpEF favorable versus unfavorable exercise PVR response sub‐groups.

Variable	Favorable PVR response (*n* = 29)	Unfavorable PVR response (*n* = 19)	*p*
Left ventricle			
LVEF (%)	61 ± 7	59 ± 6	0.73
LV Mass Indexed (g/m^2^)	57.7 ± 24.8	56.9 ± 13.7	0.89
LV EDVi (mL/m^2^)	65.8 ± 15.3	73.1 ± 30.6	0.34
LV ESVi (mL/m^2^)	30.8 ± 10.5	38.2 ± 29.4	0.30
LV strain longitudinal (%)	−10.4 ± 3.6	−10.4 ± 3.9	0.98
LV strain circumferential (%)	−15.0 ± 3.5	−14.1 ± 3.8	0.41
LV strain radial (%)	24.4 ± 7.7	22.4 ± 7.4	0.38
Right ventricle			
RVEF (%)	47 ± 11	45 ± 10	0.46
RV EDVi (mL/m^2^)	76.9 ± 19.1	80.6 ± 24.4	0.58
RV ESVi (mL/m^2^)	42.0 ± 16.6	46.4 ± 20.4	0.44
RV strain longitudinal (%)	−14.2 ± 5.3	−14.9 ± 5.8	0.69
RV strain circumferential (%)	−11.9 ± 3.1	−12.2 ± 3.3	0.79
RV strain radial (%)	18.8 ± 6.3	19.2 ± 5.7	0.84
Atrial data			
LA EF (%)	42 ± 19	39 ± 14	0.49
LA EDVi (mL/m^2^)	42.5 ± 21.6	39.7 ± 25.6	0.68
LA ESVi (mL/m^2^)	27.3 ± 21.5	25.9 ± 21.8	0.82
LA longitudinal strain (%)	20.3 ± 11.7	16.2 ± 8.8	0.18
LA strain rate (s^−1^)	0.90 ± 0.94	0.43 ± 0.37	0.02
RA EF (%)	38 ± 17	39 ± 14	0.81
RA EDVi (mL/m^2^)	29.7 ± 26.3	29.0 ± 16.7	0.91
RA ESVi (mL/m^2^)	17.4 ± 9.3	18.0 ± 9.8	0.20
RA longitudinal strain (%)	18.2 ± 12.3	16.0 ± 8.0	0.53
RA strain rate (s^−1^)	0.91 ± 0.83	0.88 ± 0.64	0.91
Others			
Epicardial fat (mL)	54.0 ± 34.0	46.2 ± 17.7	0.35
Septal angle (°)	144 ± 15	142 ± 15	0.57
Hemodynamics in subgroup with cardiac MRI data			
PVR at rest (wood units)	2.7 ± 1.7	3.6 ± 2.2	0.13
PVR at exercise (wood units)	1.9 ± 1.2	4.0 ± 3.2	0.01
∆PVR, %	−26 ± 25	+10 ± 29	< 0.001
IpcPH/CpcPH	14/15	3/16	

Abbreviations: CpcPH, combined pre‐ and post‐capillary pulmonary hypertension; IpcPH, isolated post‐capillary pulmonary hypertension; LA EDVi, left atrial end‐diastolic volume index; LA ESVi, left atrial end‐systolic volume index; LA, left atrium; LAEF, left atrial ejection fraction; LV EDVi, Left Ventricular End‐Diastolic Volume Index; LV ESVi, Left ventricular end‐systolic volume index; LV mass indexed, left ventricular mass indexed; LV mass, left ventricular mass; LV, left ventricle; LVEF, Left ventricular ejection fraction; PVR, pulmonary vascular resistance; RA EDVi, right atrial end‐diastolic volume index; RA ESVi, right atrial end‐systolic volume index; RA, right atrium; RAEF, right atrial ejection fraction; RV EDVi, right ventricular end‐diastolic volume index; RV ESVi, right ventricular end‐systolic volume index; RV, right ventricle; RVEF, right ventricular ejection fraction.

### 3.6 Regression‐Based Correlates of *Unfavorable* Exercise PVR in HFpEF: Atrial Fibrillation, COPD and Left Atrial Enlargement

As shown in Table 4, atrial fibrillation was the strongest correlate of unfavorable exercise PVR response in HFpEF (β‐estimate = 1.56 [SE 0.49], *p* = 0.001), while COPD was the only other significant risk factor (β‐estimate = 1.79 [SE 0.71], *p* = 0.01). Among echocardiographic metrics, left atrial enlargement (increased LAVI, β‐estimate = 0.05 [SE 0.02], *p* = 0.045), RV:PA uncoupling (TAPSE/PASP, β‐estimate = −3.58 [SE 1.75], *p* = 0.04), and RV function (TAPSE, β‐estimate = −0.11 [SE 0.05], *p* = 0.03) were significant correlates.

**TABLE 4:** Correlates of unfavorable exercise PVR response in HFpEF.

Variables	β‐coefficient	Standard error	OR	OR confidence interval	*p*
Univariate logistic regression					
Age	0.033	0.02	1.03	0.99, 1.08	0.09
Female sex	0.057	0.45	1.06	0.43, 2.58	0.90
BMI	−0.03	0.03	0.97	0.92, 1.03	0.36
H_2_FPEF score	0.23	0.13	1.26	0.98, 1.65	0.08
Comorbidities					
Diabetes	−0.01	0.48	0.99	0.38, 2.54	0.98
HTN	0.12	0.69	1.13	0.29, 4.73	0.86
COPD	1.79	0.71	5.97	1.65, 28.59	**0.01**
Atrial fibrillation	1.56	0.49	4.78	1.88, 12.83	**0.001**
Scleroderma	−0.11	0.94	0.90	0.11, 5.70	0.91
Echocardiogram					
LVMI	−0.005	0.006	0.995	0.98, 1.01	0.46
LAVI	0.05	0.02	1.05	1.00, 1.1	**0.045**
E/E' average	0.002	0.04	1.00	0.91, 1.08	0.96
TAPSE	−0.11	0.05	0.89	0.80, 0.98	**0.03**
TAPSE/PASP	−3.58	1.75	0.03	0.00058, 0.65	**0.04**
Epicardial fat	0.02	0.09	1.02	0.85, 1.24	0.81
Cardiopulmonary exercise test					
Peak VO_2_	−0.07	0.05	0.93	0.83, 1.03	0.18
Peak O_2_ pulse	0.01	0.046	1.01	0.91,1.12	0.83
V_E_/VCO_2_ slope	−0.005	0.026	0.99	0.94,1.05	0.84
Multivariate logistic regression models					
Model#1 clinical data					
Intercept	−1.60	2.78	0.20	(0.001, 44.0)	0.56
Age	−0.005	0.03	0.99	(0.94, 1.05)	0.85
Female sex	0.38	0.57	1.47	(0.49, 4.58)	0.50
BMI	0.006	0.04	1.01	(0.93, 1.09)	0.89
COPD	1.74	0.81	5.71	(1.27, 33.0)	**0.03**
Atrial fibrillation	1.93	0.65	6.91	(2.07, 27.1)	**0.003**
Model#2 Clinical + echocardiogram data					
Intercept	−4.54	2.66	0.011	(0, 1.15)	0.09
Age	0.02	0.05	1.02	(0.93, 1.14)	0.63
COPD	3.26	1.42	26.1	(2.4834.4)	**0.02**
Atrial fibrillation	1.60	0.87	4.94	(0.96, 32.1)	**0.06**
LAVI	0.05	0.03	1.06	(0.99, 1.14)	0.09
TAPSE	0.03	0.08	1.03	(0.88, 1.21)	0.70

*Note:* Significant *p* values < 0.05 are bolded.

Abbreviations: BMI, body mass index; COPD, chronic obstructive pulmonary disease; CPET, cardiopulmonary exercise test; E/E', ratio of early diastolic mitral blood flow velocity to mitral annulus velocity; HTN, hypertension; LAVI, left atrial volume index; LVMI, left ventricular mass index; OR, odds ratio; PASP, pulmonary arterial systolic pressure; TAPSE, tricuspid annular plane systolic excursion; V_E_/VCO_2_, minute ventilation/carbon dioxide production; VO_2_, oxygen consumption.

The first multivariate logistic regression model including biological variables (age, sex, BMI) and significant clinical variables (atrial fibrillation and COPD), both atrial fibrillation (β‐estimate = 1.93 [SE 0.65], *p* = 0.003) and COPD (β‐estimate = 3.26 [SE 1.42], *p* = 0.02), remained significant. However, in the second multivariate regression model, replacing sex and BMI with significant echocardiographic metrics of LA size (LAVI) and RV function (TAPSE), only COPD remained significant (β‐estimate = 3.26 [SE 1.42], *p* = 0.02), while atrial fibrillation approached significance (β‐estimate = 1.60 [SE 0.87], *p* = 0.06).

### 3.7 Validation Cohort

To validate findings of atrial fibrillation, left atrial enlargement and COPD in another cohort, we assessed 26 consecutive HFpEF participants undergoing invasive hemodynamic study, after the primary study analyses were completed (Table S1). Compared to favorable PVR HFpEF group (*n* = 18), unfavorable PVR HFpEF group (*n* = 8) had rest PVR (WU) 4.5 ± 2.5 (vs. 2.8 ± 2.1, *p* = 0.001), exercise PVR 5.0 ± 2.6 WU (vs. 2.0 ± 1.5, *p* < 0.001), and ∆PVR +12 ± 12 (vs. −23% ± 16%, *p* < 0.001), as expected from pre‐defined criteria. For physiological relevance, unfavorable PVR HFpEF subgroup trended towards poor gas exchange (peak VO_2_ 13.4 ± 2.2 mL/kg/min^−1^) (vs. 17.1 ± 5.3, *p* = 0.055), oxygen uptake slope 1.22 ± 0.34 mL/min/Log[L/min] (vs. 1.62 ± 0.58, *p* = 0.001) and V_E_/VCO_2_ slope 37.7 ± 11.9 (vs. 31.8 ± 6.1, *p* = 0.90).

While statistically insignificant (due to small sample size), there was a trend among unfavorable (vs. favorable) exercise PVR subgroup in HFpEF of an increased incidence of COPD (22% vs. 13%, *p* = 0.06), atrial fibrillation (38% vs. 17%, *p* = 0.96), increased left atrial size (LAVI: 29 ± 9 vs. 25 ± 12, *p* = 0.26) and poor RV:PA coupling (TAPSE/PASP: 0.31 ± 0.09 vs. 0.90 ± 0.39, *p* = 0.06).

## 4 Discussion

In this study, we report that the normal vasodilatory exercise response of pulmonary circulation can be impaired in different PH hemodynamic phenotypes, which likely indicate pulmonary vascular disease of differing underlying mechanisms (in HFpEF vs. pre‐capillary PH). Using a stepwise approach, we define an unfavorable PVR response with exercise as exercise PVR > 1.74 WU and ∆PVR decrease with exercise of < 22%. Our findings suggest that COPD and atrial fibrillation are two risk factors with the strongest association with pulmonary vascular disease in HFpEF. Left atrial size is an additional risk factor for pulmonary vascular disease in HFpEF, which is inter‐related to atrial fibrillation. In the unfavorable PVR HFpEF sub‐group, ventricular morphology was similar to the favorable PVR response, which highlights unique changes to pulmonary circulation due to atrial fibrillation and COPD in HFpEF patients.

Therapeutic and prognostic relevance of an impaired exercise PVR response has been reported in prior studies (Caravita et al. 2023; Shah et al. 2022; Zampierollo‐Jaramillo et al. 2024; Lewis 2010). However, defining a normal versus abnormal PVR response with exercise is complex and challenging as pulmonary vascular remodeling in different phenotypes involves varying levels of pulmonary vascular tree (e.g., arterioles in pre‐capillary PH, venules in combined pre‐ and post‐capillary PH) (Humbert et al. 2022; Fayyaz et al. 2018; Huston and Shah 2022). Hence, we took a stepwise approach to create dichotomous groups (favorable vs. unfavorable exercise PVR groups, Figure 1), which were leveraged to define high‐risk features with logistic regression analyses. Beyond identifying high risk features, our approach for defining a favorable exercise PVR response was not intended to claim definition of a ‘normal’ response. A broader consensus among expert centers is warranted for defining a normal exercise PVR response, which takes biological variables into account (age, sex) and defines PVR change with exercise on a graded exercise protocol (low‐, moderate‐, high‐workloads). Nevertheless, the findings of this study can be employed to identify groups of patients with and without pulmonary vascular disease, which can lead to targeted treatments. Specifically, in HFpEF, left‐to‐right atrial shunt device use has been reported to benefit patients without pulmonary vascular disease (Borlaug et al. 2022). Hence, patients with pulmonary vascular disease may not garner benefit from atrial shunt devices and might need to consider alternative therapies, for example, pulmonary artery dennervation (Borlaug et al. 2022; Caravita et al. 2023; Zhang et al. 2019).

The strong association of atrial fibrillation and COPD with pulmonary vascular remodeling in HFpEF provides an interesting mechanistic link for future studies. However, the influence of atrial fibrillation on pulmonary circulation is interlinked with left atrial remodeling, which is a well described and widely accepted paradigm (Huston and Shah 2022; Melenovsky et al. 2015; Gunturiz‐Beltrán et al. 2022). In a recent study of rest‐only hemodynamics, Chan and colleagues reported that in HFpEF, there was a higher incidence of atrial fibrillation and left atrial dilation in combined pre−/post‐capillary PH, compared to isolated post‐capillary PH^15^. These traditional post‐capillary PH phenotypes are defined based on steady hemodynamic afterload in pulmonary circulation (pulmonary vascular resistance) (Vanderpool et al. 2018; Caravita et al. 2023; Levine et al. 2019) faced by the right ventricle, while the pulsatile components also contribute significantly to RV afterload (pulmonary impedance, proximal artery compliance, vascular distensibility) (Tao et al. 2024; Raza, Kozitza, et al. 2022; Raza and Chesler 2023). In a healthy heart, the right ventricle has remarkable reserve under normal low pressure‐high compliance pulmonary circulation (Cornwell et al. 2020; Wright et al. 2020). During exercise, augmentation of cardiac output is accommodated with dilation of the pulmonary vasculature (recruitment of arterioles and capillaries), resulting in low hemodynamic load and normal RV reserve (Houston et al. 2023). However, in different PH phenotypes, pulmonary vasculature becomes less distensible with loss of compliance, impacting exercise capacity and gas exchange, resulting in eventual RV failure (Kozitza et al. 2022; Houston et al. 2023; Hsu et al. 2016; Malhotra, Dhakal, et al. 2016). An anatomic‐pathological study from the Mayo Clinic Registry of left heart failure‐PH showed that the pattern of pulmonary vasculature remodeling is usually more severe in veins, presenting in a way similarly to pulmonary veno‐occlusive disease (Fayyaz et al. 2018). As atrial fibrillation is considered a disease of pulmonary veins and venules, it is possible that the advanced pulmonary vascular remodeling occurs in a similar anatomical pattern and leads to increased hemodynamic pulmonary vascular load (Mahida et al. 2015; Cheniti et al. 2018). Similarly, pulmonary vascular remodeling is well described in COPD (Kovacs et al. 2018; Sakao et al. 2014) and with an increasing co‐incidence of HFpEF and COPD, extra attention should be paid on the interplay of these two comorbidities on pulmonary circulation. While more research is needed to define the pulmonary vascular pathobiology of atrial fibrillation and COPD in HFpEF, our current findings can help target appropriate treatments in such patients with possible interventions including anti‐fibrotic medications, catheter ablation for atrial fibrillation or pulmonary artery denervation catheter procedure (Zhang et al. 2019; Joshi et al. 2023; Chieng et al. 2023).

Notably, these risk factors of atrial fibrillation and COPD are uniquely different than typical cardiometabolic risk factors in HFpEF (obesity, diabetes, sleep apnea) (Peters et al. 2022; Dixon et al. 2023). Unlike prior HFpEF phenomapping studies that primarily cluster patients by cardiometabolic characteristics, thereby generating phenogroups with distinct comorbidity profiles (Peters et al. 2022; Dixon et al. 2023), our physiology‐based classification focuses on exercise pulmonary vascular reserve to identify determinants of pulmonary vascular remodeling. Consequently, overlap in traditional HFpEF risk factors is expected, as patient classification is driven by predefined exercise pulmonary vascular physiology, rather than underlying comorbidity burden. Our study emphasizes the impact of comorbidities of atrial fibrillation and COPD on impairments of pulmonary vascular‐left atrial axis, which is further supported by shared biventricular morphology per cardiac MRI data. In addition to pathobiological signatures, future studies are needed to also develop new structural and flow dynamic vessel‐specific models of pulmonary vascular remodeling in HFpEF with advanced imaging techniques, for example, 4D flow MRI and cardiac positron emission tomography scan (Bissell et al. 2023; Wieben et al. 2023; Barton et al. 2019).

### 4.1 Limitations

This is a single‐center, retrospective study and warrants future validation across larger multi‐center cohorts. In larger studies, the clinical relevance of the proposed PVR exercise response needs to be validated in correlating with clinical adverse outcomes (e.g., mortality, hospitalization) and change in these exercise PVR responses post‐treatment. The non‐cardiac dyspnea group does not represent an entirely healthy population since they underwent invasive cardiopulmonary testing, given the initial concern for dyspnea.

### 4.2 Conclusions

Pulmonary vascular remodeling and an impaired exercise PVR response occur in different PH phenotypes, including HFpEF. While COPD and atrial fibrillation are the strongest risk factors for an unfavorable exercise PVR response, the influence of atrial fibrillation is inter‐linked to left atrial myopathy. The ventricular morphology of HFpEF patients is similar among favorable versus unfavorable exercise PVR sub‐groups, which speaks to a unique abnormality related to pulmonary vasculature‐left atrial axis in HFpEF. While these findings can help identify patients at higher risk of severe pulmonary vascular remodeling in different PH phenotypes, additional mechanistic studies are warranted to define causal behaviors.

## Funding

This work was supported by American Heart Association, 23CDA1057697. National Institutes of Health, KL2TR002374‐07.

## Conflicts of Interest

The authors declare no conflicts of interest.

## Supporting information


**Table S1:** Validation cohort.
**Figure S1:** One‐year survival from mortality and heart failure hospitalizations among two HFpEF subgroups with favorable vs. unfavorable exercise PVR response.

## Data Availability

The data that support the findings of this study are available from the corresponding author upon reasonable request.
