# Supplementary material for: Determinants of Abnormal Pulmonary Vasodilatory Response With Exercise in HFpEF: Pulmonary Vascular‐Left Atrial Axis Abnormalities
Source: Compr Physiol. 2026 Jul 26;16(4):e70229. doi: 10.1002/cph4.70229 (PMC13402025; doi:10.1002/cph4.70229)
Supplement: Supplementary file 1 — Table S1: Validation cohort. Figure S1: One‐year survival from mortality and heart failure hospitalizations among two HFpEF subgroups with favorable vs. unfavorable exercise PVR response. [file CPH4-16-e70229-s001.docx]

**Supplemental data:**

| **Variables** | **Favorable PVR HFpEF group**  n=18 | **Unfavorable PVR HFpEF group**  n=8 | **p-value** |
| --- | --- | --- | --- |
| **Age** (years) | 67 ± 13 | 68 ± 13 | 0.10 |
| **Female Sex**, n (%) | 11 (61) | 7 (88) | 0.88 |
| **BMI** (Kg/m^2^) | 35 ± 6 | 38 ± 11 | 0.04 |
| **BNP** (pg/mL) | 197± 322 | 129 ± 87 | 0.002 |
| **6MWD** (meter) | 295 ± 111 | 178 ± 90 | 0.002 |
| **Comorbidities** | | | |
| Diabetes, n (%) | 9 (50) | 3 (38) | 0.95 |
| HTN, n (%) | 16 (89) | 5 (63) | 0.58 |
| COPD, n (%) | 1 (13) | 4 (22) | 0.06 |
| CAD, n (%) | 6 (33) | 4 (50) | 0.33 |
| Atrial Fibrillation, n (%) | 3 (17) | 3 (38) | 0.96 |
| Scleroderma, n (%) | 3 (17) | 0 (0) | - |
| OSA, n (%) | 10 (56) | 5 (63) | 0.81 |
| **Echocardiogram** | | | |
| LVEF (%) | 62 ± 5 | 64 ± 4 | 0.92 |
| LVMI (g/m²) | 77.4 ± 20.8 | 66.1 ± 13.8 | 0.35 |
| LAVI (mL/m²) | 25.2 ± 12.5 | 29.0 ± 8.9 | 0.26 |
| E/E’ Average | 12.3 ± 4.8 | 9.9 ± 2.5 | 0.64 |
| TAPSE/PASP (mm/mm Hg) | 0.90 ± 0.39 | 0.31 ± 0.09 | 0.06 |
| Epicardial fat (mm) | 6.0 ± 1.4 | 6.0 ± 4.2 | 0.72 |
| **Rest Hemodynamics:** | | | |
| Heart rate (beats/min) | 72 ± 11 | 72 ± 12 | 0.83 |
| Mean AO (mmHg) | 110 ± 16 | 102 ± 18 | 0.60 |
| RA (mmHg) | 14 ± 5.1 | 13 ± 4.9 | 0.95 |
| mPAP (mmHg) | 32 ± 12 | 41 ± 10 | **0.006** |
| PAWP (mmHg) | 19 ± 4 | 18 ± 2.8 | 0.43 |
| Direct Fick CO (L/min) | 5.7± 1.2 | 5.5 ± 1.3 | **0.003** |
| SVI (mL/m^2^) | 38 ± 6 | 40 ± 11 | 0.14 |
| PVR (woods units) | 2.8 ± 2.1 | 4.5 ± 2.5 | **0.001** |
| **Exercise Hemodynamics:** | | | |
| Heart rate (beats/min) | 101± 20 | 96 ± 19 | 0.52 |
| mPAP (mmHg) | 48 ± 13 | 64 ± 10 | **<0.001** |
| PAWP (mmHg) | 30 ± 5.9 | 28 ± 7.1 | 0.107 |
| Direct Fick CO (L/min) | 10.6 ± 2.4 | 8.2 ± 2.3 | **<0.001** |
| SVI (mL/m^2^) | 48.6 ± 10.6 | 43.6 ± 13.1 | **<0.001** |
| PVR (woods units) | 2.0 ± 1.5 | 5.0 ± 2.6 | **<0.001** |
| ∆ PVR | -0.24 ± 1.03 | +0.30 ± 0.67 | **<0.001** |
| PVR change (%) | -23 ± 16 | +12 ± 12 | **<0.001** |
| mPAP/CO slope | 4.9 ± 3.9 | 13.3 ± 7.8 | **<0.001** |
| PAWP/CO slope | 4.2 ± 4.0 | 7.9 ± 7.7 | 0.39 |
| **Cardiopulmonary Exercise Test:** | | | |
| Peak VO_2_  (mL/Kg.min^-1^) | 17.1 ± 5.3 | 13.4 ± 2.2 | 0.055 |
| Peak VO_2_ %predicted | 74 ± 25 | 52 ± 11 | 0.62 |
| OUES (mL/min/Log[L/min]) | 1.62 ± 0.58 | 1.22 ± 0.34 | **0.001** |
| V_E_/VCO_2_ slope | 31.8 ± 6.1 | 37.7 ± 11.9 | 0.90 |

**Supplemental Table 1 Validation cohort**

Abbreviations: BMI: Body Mass Index, BNP: B-type Natriuretic Peptide, 6MWD: 6-Minute Walk Distance, HTN: Hypertension, COPD: Chronic Obstructive Pulmonary Disease, CAD: Coronary Artery Disease, CTD: Connective Tissue Disease, OSA: Obstructive Sleep Apnea, LVEF: Left Ventricular Ejection Fraction, LVMI: Left Ventricular Mass Index, LAVI: Left Atrial Volume Index, E/E’: Ratio of Early Diastolic Mitral Inflow Velocity to Mitral Annular Early Diastolic Velocity, TAPSE/PASP: Tricuspid Annular Plane Systolic Excursion / Pulmonary Artery Systolic Pressure, Mean AO: Mean Aortic Pressure, RA: Right Atrial Pressure, mPAP: Mean Pulmonary Artery Pressure, PAWP: Pulmonary Artery Wedge Pressure, Direct Fick CO: Direct Fick Cardiac Output, SVI: Stroke Volume Index, PVR: Pulmonary Vascular Resistance, mPAP/CO: Mean Pulmonary Artery Pressure to Cardiac Output Ratio, PAWP/CO: Pulmonary Artery Wedge Pressure to Cardiac Output Ratio.


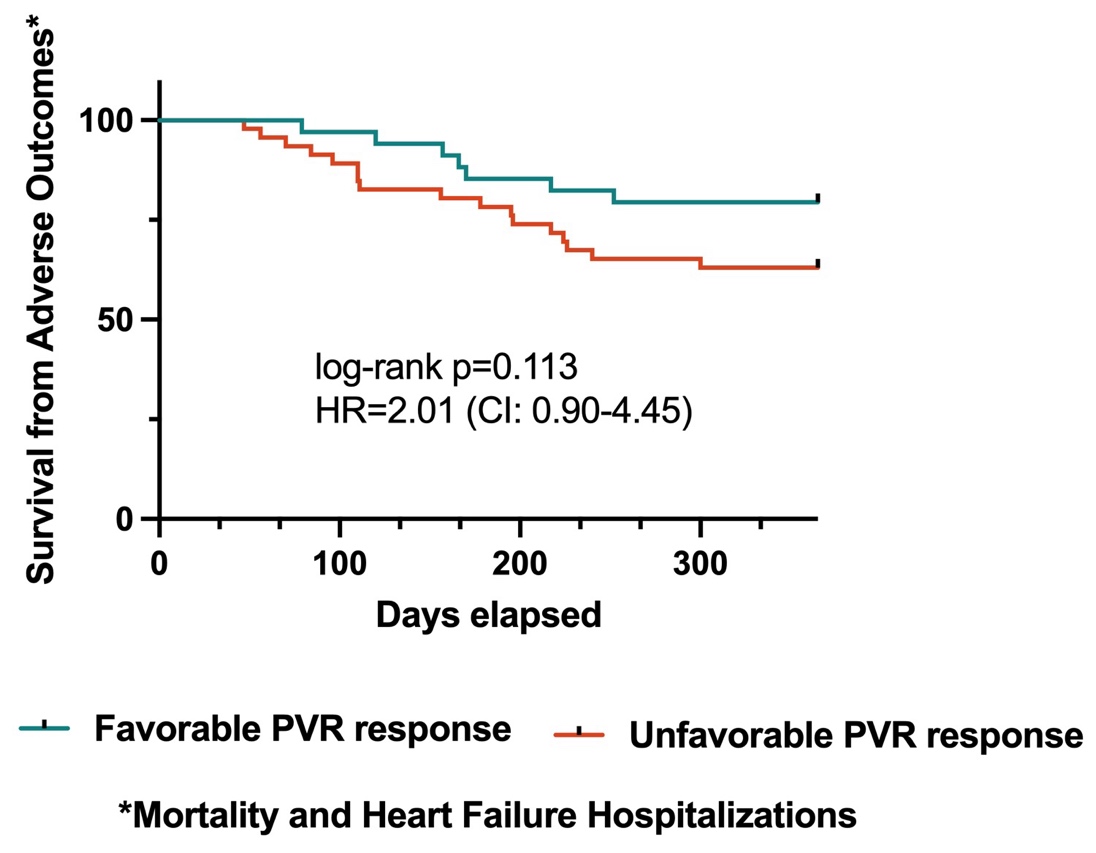


**Supplemental Figure 1. One-year survival from mortality and heart failure hospitalizations among two HFpEF subgroups with favorable vs unfavorable exercise PVR response.**
